# Incorporating support vector machine with sequential minimal optimization to identify anticancer peptides

**DOI:** 10.1186/s12859-021-03965-4

**Published:** 2021-05-29

**Authors:** Yu Wan, Zhuo Wang, Tzong-Yi Lee

**Affiliations:** 1 School of Life and Health Sciences, The Chinese University of Hong Kong, Shenzhen, Shenzhen, 518172 Guangdong People’s Republic of China; 2 Warshel Institute for Computational Biology, The Chinese University of Hong Kong, Shenzhen, Shenzhen, 518172 Guangdong People’s Republic of China

**Keywords:** Anticancer peptides, SVM, SMO, PSSM

## Abstract

**Background:**

Cancer is one of the major causes of death worldwide. To treat cancer, the use of anticancer peptides (ACPs) has attracted increased attention in recent years. ACPs are a unique group of small molecules that can target and kill cancer cells fast and directly. However, identifying ACPs by wet-lab experiments is time-consuming and labor-intensive. Therefore, it is significant to develop computational tools for ACPs prediction. Though some ACP prediction tools have been developed recently, their performances are not well enough and most of them do not offer a function to distinguish ACPs from antimicrobial peptides (AMPs). Considering the fact that a growing number of studies have shown that some AMPs exhibit anticancer function, this work tries to build a model for distinguishing AMPs from ACPs in addition to a model that predicts ACPs from whole peptides.

**Results:**

This study chooses amino acid composition, N5C5, k-space, position-specific scoring matrix (PSSM) as features, and analyzes them by machine learning methods, including support vector machine (SVM) and sequential minimal optimization (SMO) to build a model (model 2) for distinguishing ACPs from whole peptides. Another model (model 1) that distinguishes ACPs from AMPs is also developed. Comparing to previous models, models developed in this research show better performance (accuracy: 85.5% for model 1 and 95.2% for model 2).

**Conclusions:**

This work utilizes a new feature, PSSM, which contributes to better performance than other features. In addition to SVM, SMO is used in this research for optimizing SVM and the SMO-optimized models show better performance than non-optimized models. Last but not least, this work provides two different functions, including distinguishing ACPs from AMPs and distinguishing ACPs from all peptides. The second SMO-optimized model, which utilizes PSSM as a feature, performs better than all other existing tools.

## Background

Cancer is a leading cause of death and the most important barrier to increasing life expectancy worldwide in this century [1]. This disease is caused by the growth and uncontrolled proliferation of abnormal cells. Conventional cancer treatments, including radiation therapy and chemotherapy, often have adverse effects on normal cells and thus not effective enough [2]. Moreover, some mechanisms also lead to drug resistance from the cancerous cells [3]. Therefore, a novel treatment that lacks adverse effects, targets specifically to cancer cells, and with a low possibility of drug resistance is in need urgently.

In recent years, a new group of small peptides, ACPs, has been discovered that can target and kill cancer cells specifically while not affecting healthy cells [4, 5]. The high selectivity and cancer-selective toxicity [6] of ACPs depend on multiple differences between cancer cells and normal cells, including membrane net charge and unique molecules on the membrane [6]. Due to their specificity and low toxicity, ACPs have attracted growing attention as a novel cancer treatment and have been considered to be promising [7]. For example, romidepsin (FK228), has been shown to have clinical effectiveness in patients with refractory cutaneous T-cell lymphoma [8]. To promote its application, it is of great significance to distinguish ACPs from all peptides. Nevertheless, finding anticancer peptides by experiments could be both time-consuming and labor-intensive [9]. To deal with this problem, computational identification before wet-lab experiments is necessary. Machine-learning-based methods could be of great help to classify and predict those special peptides. Moreover, some characteristics of cancer cells, such as the negative surface charge of their membrane, also shared by bacterial cells [10]. In fact, a hypothesis is proposed that ACPs share similar features with another group of small molecules that can specifically target and kill microbes, called AMPs [11]. Indeed, some AMPs are discovered to exhibit anticancer function according to recent studies [12]. Thus, distinguishing ACPs from AMPs may promote the discovery of ACPs more accurate, more convenient and faster.

To identify and predict ACPs, many computational tools for predicting ACPs have been designed, including Hajisharifi’s model [13], AntiCP [14], iACP [15], MLACP [16], mACPpred [17], ACPred [18], ACPred-Fuse [19] and ACPred-FL [20]. Hajisharifi et al. use physicochemical properties and PseAAC as characteristics of peptide sequences, and SVM as a machine learning method to identify ACPs. Their method is claimed to perform with an accuracy of 83.82% [13]. By analyzing the AAC of peptides and using SVM as a machine learning method, AntiCP offers two models that can distinguish ACPs from either AMPs or non-ACPs based on different datasets [14]. In MLACP, they analyze the AAC, dipeptide composition, atomic composition and physicochemical properties separately and hybridlike. Then they apply two machine learning methods: SVM and random forest to build models based on peptide characteristics. The performance of MLACP is claimed to be better than any other existing methods, with an accuracy of 87.5%. The deficiency of the MLACP study is that it does not offer a model that can distinguish ACPs from AMPs [16]. mACPpred, which achieves an accuracy of 88.5% in their independent test, uses SVM as the final classifier. ACPred also utilizes SVM and analyzes several different features, the accuracy of which is 97.56% according to their paper. ACPred-FL incorporated feature representation learning and feature selection with SVM. The prediction accuracy of this tool in their independent test is 85.7%. Similarly, ACPred-Fuse fuses a feature representation learning model that integrates 29 different features with random forest, and performs an 89% accuracy in their independent test.

This research offers more functions and better performance. First of all, sequences of examined ACPs, non-ACPs, and AMPs without anti-cancer functions are collected. With these data, two different groups of datasets are constructed: (1) inspected ACPs as positive data and AMPs without anti-cancer function as negative data; (2) examined ACPs as positive data, simple non-ACPs as negative data. Then characteristics of those peptide sequences are analyzed, considering four features, amino acids composition (AAC), N5C5, k-space and PSSM, separately and also hybridized. It is the first time that PSSM is considered as a feature in ACP prediction studies. Based on the analysis of those features, several models are built based on two machine learning methods: SVM [21] and SMO [22]. Comparing the performance of those models, two best ones are chosen: SMO-1, which utilizes SMO to analyze AAC and k-space feature of a dataset (1), and SMO-2, which uses SMO as well and is based on analysis of AAC, N5C5 and k-space of the dataset (2). At last, the same testing dataset is applied to test the performance of SMO-1, SMO-2, AntiCP-1, AntiCP-2, mACPpred, ACPred, ACPred-Fuse and ACPred-FL. As for results, comparing to AntiCP-1, which is also designed to distinguish ACPs from AMPs, SMO-1 shows higher accuracy, specificity and Matthews Correlation Coefficient (MCC). Also, the performance of SMO-1 is of better balance. As for SMO-2, identifying ACPs from all kinds of peptides, performs better with consideration of accuracy, sensitivity, specificity and MCC, and shows relatively more balanced performance than AntiCP-2, mACPpred, ACPred, ACPred-Fuse and ACPred-FL do. In general, this research built two models with different functions: one is for predicting ACPs from AMPs, which share some similarities to ACPs, and another one is used to distinguish ACPs from all peptides. The second SMO-optimized model shows better performance than the unoptimized model and other existing tools.

## Results

### Characterization of the sequence-based features of ACPs

**Fig. 1:**
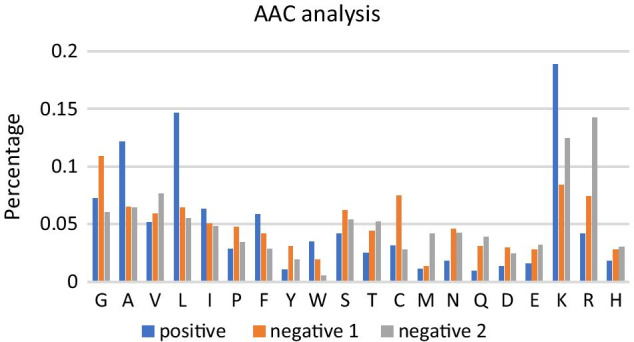
AAC analysis of positive, negative 1, negative 2. The frequency percentage of each amino acid in positive, negative1 and negative 2 data groups is shown in blue, orange and gray respectively

Comparing to AMPs but non-ACPs (peptides in negative dataset 1), K, L, A are much more frequent in ACPs, whereas N, Y, Q are dominant in negative dataset 1(with the lowest p-values). Similarly, comparing to non-ACPs in negative dataset 2, L, W, A are dominant in ACPs, whereas M, R, Q are dominant in non-ACPs (with the lowest p-values) (Fig. 1). Those significant differences in the frequency of each amino acid in different datasets contribute greatly to later classification.

Also, some of these results agree with the physicochemical properties of amino acids. For example, K is the most predominant amino acid in the positive dataset while hydrophobic positively charged lysine-rich peptides which act as cationic peptides that can indeed interact with anionic membranes on cancer cells, disrupt the cell membrane integrity, penetrate into the membrane and thus serve as ACPs [23].

**Fig. 2:**
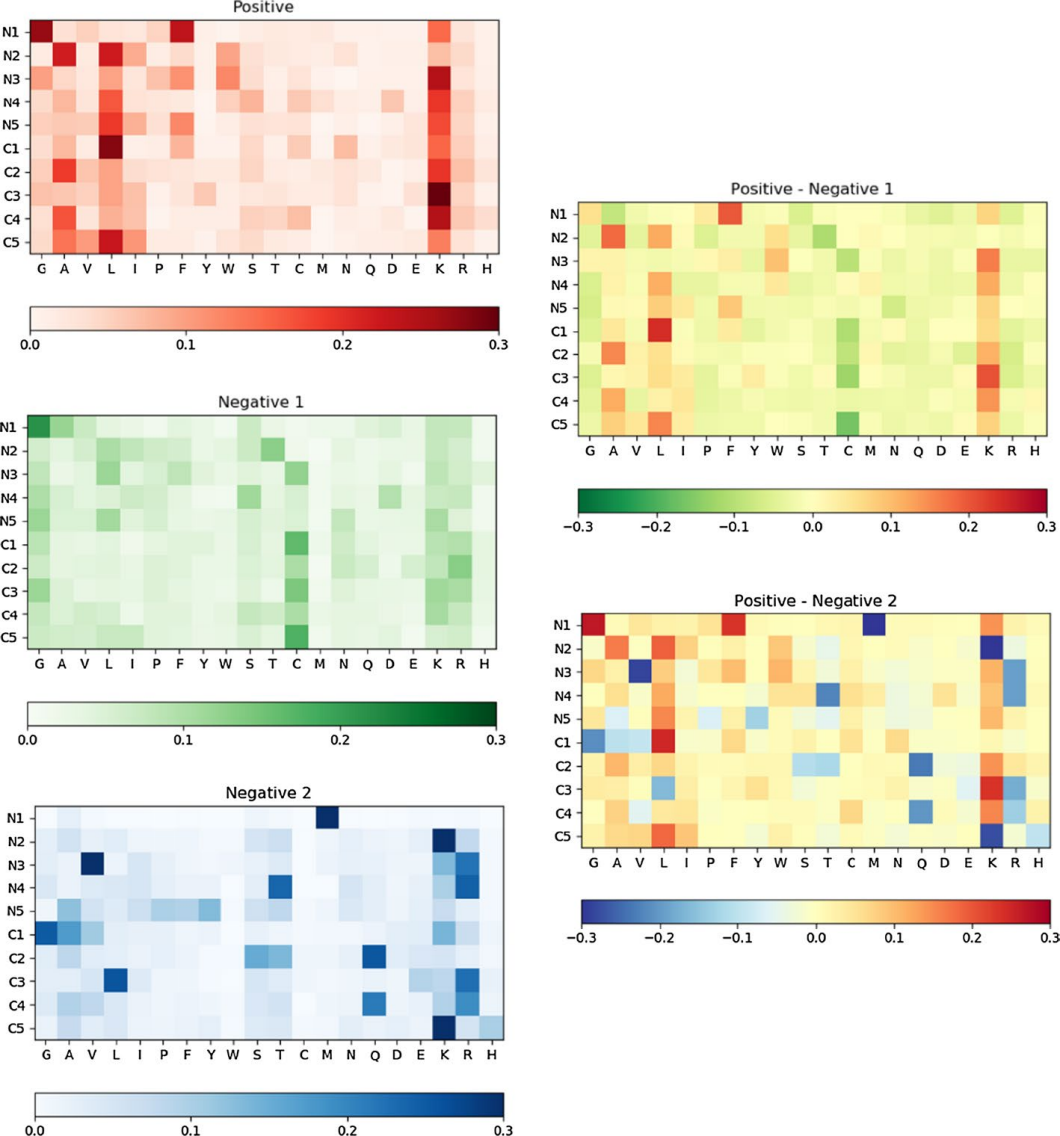
N5C5 analysis results. The three heatmaps on the left show frequency of each amino acid at each position in three different data groups, including positive, negative 1 and negative 2 data. The two heatmaps on the right show different values of each amino acid at each position comparing positive to negative 1 and negative 2 separately

According to the result of the positive dataset, K, L are the two most dominant amino acids in N5C5 of ACPs. Taking position under consideration, K is dominant in the third position of C-terminal end, L is dominant in the first position of C-terminal end, and G is dominant in the first position of N-terminal end. G is also dominant in the first position of N-terminal end of the negative 1 group (AMPs but non-ACPs). Contrarily, M is the most frequent one in the first position of N-terminal end of non-ACPs in the negative dataset 2. Comparing positive dataset to negative 1 dataset, significant differences can be found: A, L, F, K are more dominant in the positive dataset while C is more dominant in the negative 1 dataset. On the contrary, comparing positive dataset to the negative 2 dataset, distributions of each amino acid in each position are more divergent, and less contrasts could be extracted (Fig. 2).

**Fig. 3:**
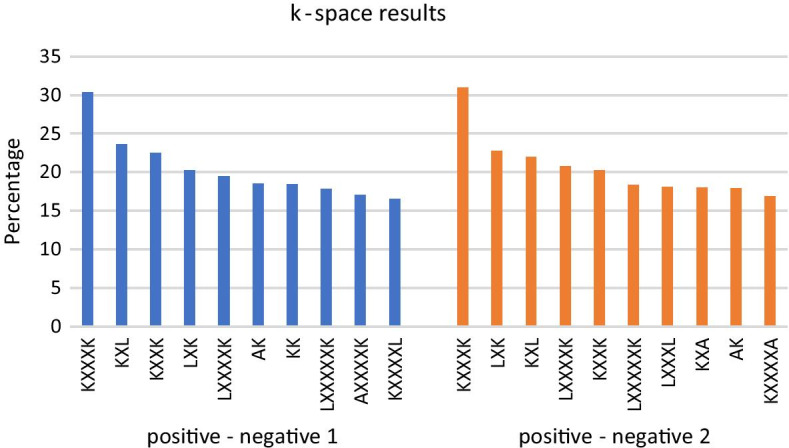
10 most different k-space pairs comparing positive to negative 1, positive to negative 2. Blue bars on the left represent the percentage of the top 10 different k-space pairs comparing positive data to negative 1 data: KXXXK, KXL, KXXK, LXK, LXXXXK, AK, KK, LXXXXXK, AXXXXK, KXXXXL. Orange bars on the right show percentage of the ten most different k-space pairs in comparison with the positive data and negative 2 data: KXXXK, LXK, KXL, LXXXXK, KXXK, LXXXXXK, LXXXL, KXA, AK, KXXXXA

Finally, with X representing spacings between amino acids, the ten most diverse k-space pairs comparing the positive data to the negative 1 data are KXXXK, KXL, KXXK, LXK, LXXXXK, AK, KK, LXXXXXK, AXXXXK, KXXXXL. The ten most different k-space pairs comparing the positive data to the negative dataset 2 are KXXXK, LXK, KXL, LXXXXK, KXXK, LXXXXXK, LXXXL, KXA, AK, KXXXXA (Fig. 3). It should be noted that these results are roughly correspondent to the previous AAC and N5C5 results.

### Model performance

Characteristics of the peptide data are then utilized to build the models, using machine learning methods such as SVM and SMO. In general, AAC, N5C5, k-space range from 0 to 2 and PSSM are used to build the model separately. Then, AAC, N5C5, k-space = 0 are hybridized in pairs and all together to build some other models. It should be noted that in SVM models (Tables 1, 2), the weight of each training model is tried to be adjusted from 0.1 to 10 and the one which could obtain the best accuracy is chosen as the final weight. First, SVM is utilized to analyze both the positive dataset and the negative dataset 1. As mentioned above, to improve the performance of those models, the weight of each model is adjusted. Among all those models, the one hybridized AAC and k-space = 0 as the feature with a weight of 0.9 performs the best, whose testing accuracy is 79.5% (Table 1).

**Table 1:** The performance of models based on both positive dataset and negative 1 dataset using SVM as classifier

Features	Training				Testing		
	Weight	Sensitivity	Specificity	Accuracy	Sensitivity	Specificity	Accuracy
AAC	0.7	0.626	0.890	0.758	0.670	0.860	0.765
N5C5	0.7	0.665	0.866	0.766	0.640	0.850	0.745
k-space = 0	1.0	0.644	0.726	0.685	0.640	0.910	0.775
k-space = 1	1.0	0.639	0.704	0.672	0.610	0.930	0.770
k-space = 2	1.0	0.641	0.737	0.689	0.600	0.910	0.755
AAC + k-space = 0	0.9	0.693	0.907	0.800	0.690	0.900	0.795
AAC + N5C5	0.5	0.645	0.950	0.798	0.640	0.890	0.765
N5C5 + k-space = 0	0.9	0.678	0.907	0.792	0.630	0.860	0.745
AAC + N5C5 + k-space 0	0.5	0.641	0.978	0.810	0.610	0.910	0.760
PSSM	0.6	0.737	0.896	0.816	0.69	0.86	0.775

**Table 2:** Performance of models based on both positive dataset and negative 2 dataset using SVM as classifier

Features	Training				Testing		
	Weight	Sensitivity	Specificity	Accuracy	Sensitivity	Specificity	Accuracy
AAC	0.6	0.873	0.952	0.913	0.860	0.942	0.928
N5C5	1.9	0.942	0.909	0.925	0.960	0.878	0.891
k-space = 0	0.4	0.799	0.957	0.878	0.750	0.952	0.918
k-space = 1	0.4	0.810	0.948	0.879	0.700	0.934	0.895
k-space = 2	0.5	0.840	0.957	0.898	0.720	0.958	0.918
AAC + k-space = 0	0.9	0.868	0.920	0.894	0.860	0.914	0.905
AAC + N5C5	0.4	0.892	0.972	0.932	0.940	0.952	0.950
N5C5 + k-space = 0	0.5	0.857	0.957	0.907	0.890	0.930	0.923
AAC + N5C5 + k-space 0	1.0	0.909	0.909	0.909	0.930	0.900	0.905
PSSM	0.7	0.909	0.911	0.913	0.910	0.838	0.850

Similarly, SVM is used to analyze both positive dataset and negative dataset 2. After adjusting the weight of each model, the one with the highest testing accuracy which hybridizes AAC and N5C5 for analysis is chosen. With a weight of 0.4, the accuracy of that model reaches 95% (Table 2). Then SMO algorithm is used to compare positive dataset to negative dataset 1. Using AAC and k-space as the representative characteristics of peptides, the accuracy of that model ranks the top one among all the models in this group at 85.5% (Table 3). Finally, models are constructed using SMO as a machine learning method and negative dataset 2 as negative data. After the evaluation of performance, the model which hybridizes AAC, N5C5 and k-space shows the highest accuracy of 95.2% (Table 4).

**Table 3:** Performance of models based on both positive dataset and negative 1 dataset using SMO as classifier

Features	Training				Testing			
	Sensitivity	Specificity	Accuracy	MCC	Sensitivity	Specificity	Accuracy	MCC
AAC	0.756	0.888	0.822	0.587	0.760	0.840	0.800	0.556
N5C5	0.700	0.808	0.754	0.511	0.660	0.820	0.740	0.486
k-space = 0	0.790	0.838	0.814	0.629	0.830	0.860	0.845	0.690
k-space = 1	0.834	0.868	0.851	0.702	0.830	0.860	0.845	0.690
k-space = 2	0.812	0.877	0.844	0.690	0.770	0.800	0.785	0.570
AAC + k-space = 0	0.840	0.793	0.816	0.634	0.850	0.860	0.855	0.710
AAC + N5C5	0.728	0.873	0.800	0.607	0.720	0.860	0.790	0.586
N5C5 + k-space = 0	0.784	0.834	0.809	0.618	0.820	0.840	0.830	0.660
AAC + N5C5 + k-space 0	0.793	0.849	0.821	0.642	0.830	0.860	0.845	0.690
PSSM	0.844	0.862	0.853	0.706	0.850	0.800	0.825	0.651

**Table 4:** Performance of models based on both positive dataset and negative 2 dataset using SMO as classifier

Features	Training				Testing			
	Sensitivity	Specificity	Accuracy	MCC	Sensitivity	Specificity	Accuracy	MCC
AAC	0.896	0.931	0.914	0.828	0.930	0.932	0.932	0.786
N5C5	0.905	0.931	0.918	0.836	0.960	0.914	0.922	0.772
k-space = 0	0.890	0.929	0.909	0.819	0.940	0.944	0.943	0.819
k-space = 1	0.933	0.950	0.942	0.884	0.910	0.944	0.940	0.803
k-space = 2	0.924	0.942	0.933	0.866	0.910	0.956	0.948	0.825
AAC + k-space = 0	0.892	0.942	0.917	0.835	0.960	0.942	0.945	0.828
AAC + N5C5	0.918	0.920	0.919	0.838	0.960	0.946	0.948	0.836
N5C5 + k-space = 0	0.922	0.948	0.935	0.871	0.970	0.948	0.950	0.843
AAC + N5C5 + k-space 0	0.927	0.950	0.938	0.877	0.970	0.948	0.952	0.847
PSSM	0.950	0.948	0.949	0.898	0.940	0.930	0.932	0.789

Among all models, two models with the best performance are chosen as the final models of this research: using SMO method to analyze AAC and k-space = 0 feature of the positive dataset against the negative 1 dataset (named as SMO-1) and using SMO method to analyze AAC, N5C5 and k-space = 0 feature of the positive dataset against the negative dataset 2 (named as SMO-2).

### Comparison with existing ACPs prediction tools in terms of performance

**Fig. 4:**
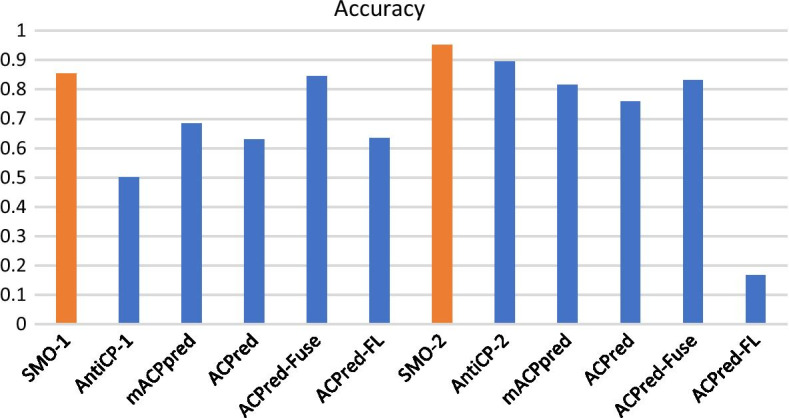
Accuracy comparison of SMO-1 (highlighted in orange), SMO-2 (highlighted in orange) and mACPpred, ACPred, ACPred-Fuse and ACPred-FL

To show the significance and success of those two models, the testing dataset is also applied to test the existing models, including the two AntiCP models, mACPpred, ACPred, ACPred-Fuse and ACPred-FL. Testing data from positive and negative 1 dataset are applied on SMO-1, AntiCP-1, mACPpred, ACPred, ACPred-Fuse and ACPred-FL. The model constructed in this work, SMO-1, shows the highest accuracy and MCC (Table 5). Although AntiCP-1 performs with the highest sensitivity and ACPred-Fuse performs with the highest specificity in all models, SMO-1 performs a more balanced result. Similarly, testing data from the positive and negative 2 datasets are applied on SMO-2, AntiCP-2, mACPpred, ACPred, ACPred-Fuse and ACPred-FL, which are all models utilized to distinguish ACPs from all kinds of peptides. Considering accuracy, sensitivity, specificity and MCC, SMO-2 shows the best performance among all models (Table 5). In general, SMO-2 performs with the highest accuracy comparing to existing models (Fig. 4).

**Table 5:** Comparison of my models and some existing tools

Datasets	Tool	Sensitivity	Specificity	Accuracy	MCC
Positive + negative 1	SMO-1	0.850	0.860	0.855	0.710
	AntiCP-1	1	0	0.500	–
	mACPpred	0.95	0.42	0.685	0.436
	ACPred	0.930	0.330	0.630	0.325
	ACPred-Fuse	0.820	0.870	0.845	0.691
	ACPred-FL	0.88	0.39	0.635	0.310
Positive + negative 2	SMO-2	0.970	0.948	0.952	0.847
	AntiCP-2	0.91	0.88	0.895	0.790
	mACPpred	0.949	0.790	0.815	0.583
	ACPred	0.930	0.724	0.758	0.501
	ACPred-Fuse	0.820	0.836	0.833	0.549
	ACPred-FL	0.880	0.024	0.167	− 0.183

## Discussion

Some problems of conventional anticancer treatments, such as drug resistance and toxicity to other normal cells, make it necessary and urgent to discover other novel anticancer treatments [2, 3]. Among those promising treatments, anticancer peptides have attracted broad attention and interest. Due to the special structure of ACPs and its specific interaction with cancer cells, this special group of molecules can target and kill cancer cells without destroying other normal cells [4]. Before the wet-lab experiment, a computational predictive tool will definitely be helpful for the identification of ACPs. Moreover, in consideration of the similarity between ACPs and AMPs [8], it is regarded as a more efficient way of ACPs identification by searching from AMPs, because there are more examined sequence data of AMPs which could be obtained.

Nevertheless, most of the existing tools only provide a function that identifies ACPs from all kinds of peptides [13–16]. Therefore, this work creates a tool with more functions and better performance of prediction. To achieve this goal, several innovative efforts or improvements have been made. In this study, we create tools with ACPs comparing to previous studies. In total, 1492 positive and 7068 negative (4433 for negative 1 and 2635 for negative 2) data are gathered from seven different sources. Then, balanced datasets with 463 sequences in each training dataset and 100 sequences, which are independent of training data, in each testing dataset are constructed. Another improvement in this research is that new features are chosen for characterization, including N5C5, k-space and PSSM. The hybridization of some of those features greatly enhances the performance.

In the model construction step, a better machine-learning algorithm, SMO [22], is chosen and applied for classification, and increases the accuracy by 7.55% and 0.2% comparing to the SVM models. This performance suggests that SMO is a better choice than SVM in this case, and shows the success of SMO in text classification, proteomics projects, and analysis of high-dimensional data. Models built in this research is further compared with previous ACP prediction tools using an independent testing dataset. SMO-1 performs better than other tools considering accuracy and MCC value, and shows more balanced results. As for SMO-2, it performs better than all other tools in general.

Even though most of the accessible data of examined ACPs are collected in this study, the amount is still not adequate. As a result, this research may have some limitation, and could be improved in the future with more sequence data.

## Conclusions

This research presents a new scheme for the identification of ACPs, including utilizing a new important feature, PSSM, and a new helpful algorithm, SMO, for optimizing SVM for classification. Also, this work offers two functions: (1) distinguishing ACPs from AMPs and (2) distinguishing ACPs from all kinds of peptides. With the help of SMO, optimized models perform better than ordinary models and other existing tools.

## Methods

**Fig. 5:**
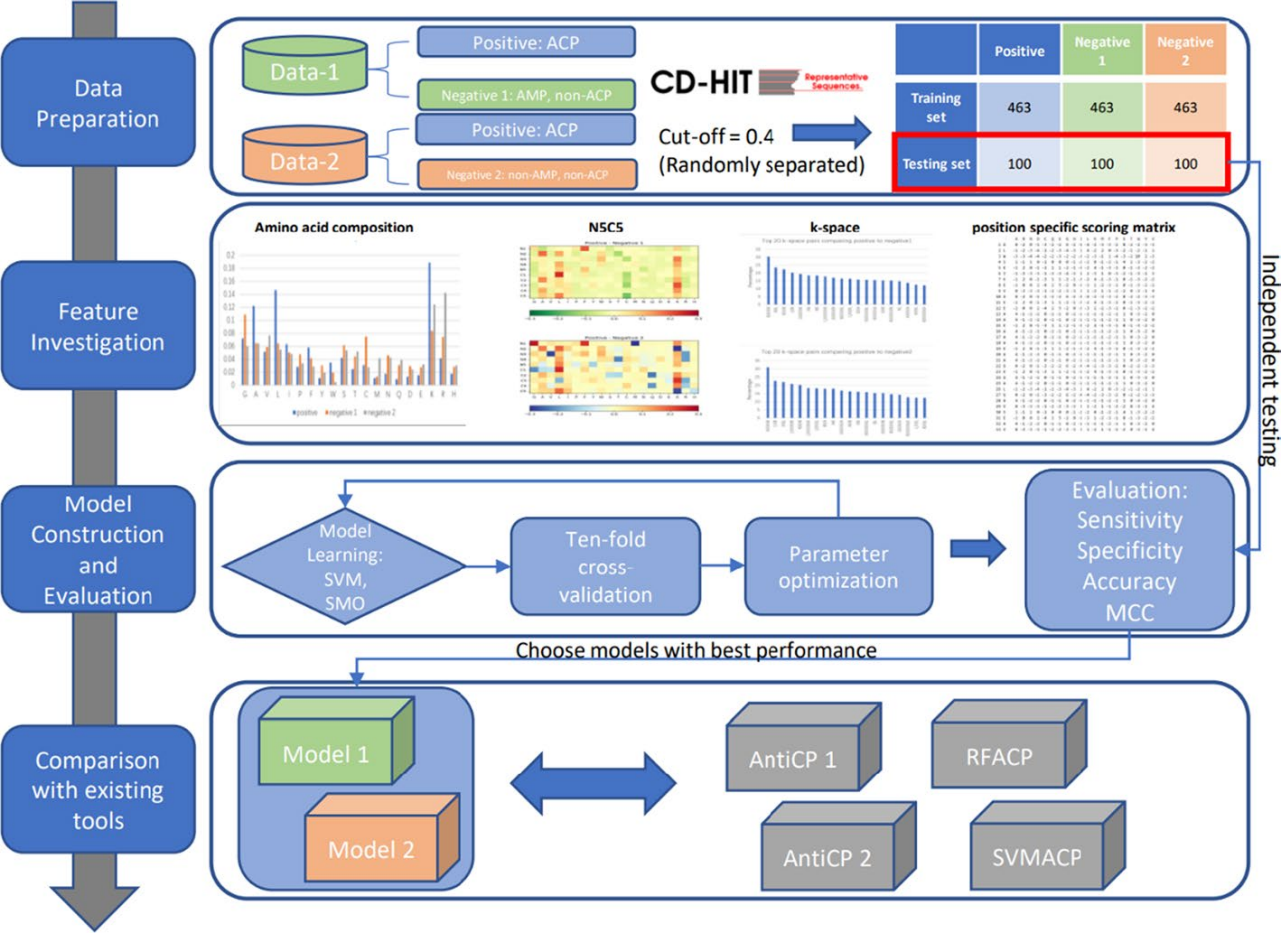
Flowchart of this work. Four major steps are involved: data preparation, feature investigation, model construction and evaluation, and comparison with existing tools. In the first step, data preparation, two datasets are constructed, which are then separated into training and testing data after CD-HIT. Then four features are investigated: amino acid composition, N5C5, k-space and position-specific scoring matrix. The third step involves model learning, cross-validation, parameter optimization and evaluation. Finally, two models proposed are compared with other existing tools

The process of this research is extracted and shown as a flowchart in Fig. 5. Details of the process will be explained in the following sections.

### Dataset preparation

In this research, three datasets are constructed: the positive dataset, negative dataset 1 and negative dataset 2. A positive dataset refers to anticancer peptides that are examined by experiments. They are collected from LEE dataset (total: 422) [16], Tyagi dataset (total: 450) [14], APD (total: 225) [24] and CancerPPD (total: 422) [25]. Negative dataset 1 is a collection of anti-microbial peptides without anti-cancer function. They are adapted from dbAMP dataset (total: 4057) [26] and Tyagi dataset (total: 1372). Peptides in negative dataset 2 are peptides without anti-microbial nor anti-cancer functions, which are collected from UniProt (total:281,665). Since anticancer peptides have been proved to be effective small molecules (< 50 amino acids) [27], peptides longer than 50 amino acids are removed out of datasets. Also, peptide contains artificial amino acids are removed. After this filtration step, 1492 peptide sequences in the positive dataset, 4433 peptide sequences in negative dataset 1 and 2635 peptide sequences in negative dataset 2 are obtained. To reduce identical or similar peptides sequence, CD-HIT program [28] is utilized in this research.

100% sequence-identity cut-off is applied on positive and negative 1 datasets using a Python program. Then the processed positive dataset is compared with processed negative dataset 1 using CD-HIT-2D [28]. It identifies and removes sequences in negative datasets that are similar to ones in positive dataset above a threshold of 40% (Table 6). To balance datasets, some of the peptide sequences in negative 1 dataset are removed randomly. Ultimately, both positive and negative 1 datasets have 563 peptide sequences. Each dataset is then divided randomly into two subsets, the one that contained 463 peptides is utilized as a training dataset and the other one which contained 100 peptides is used as a testing dataset. Considering the fact that normal peptides are much more abundant than ACPs in nature, the negative dataset 2 is constructed with 963 peptides collected randomly from the original 2635 peptides, and then be randomly divided into positive and negative dataset 2, with 463 and 500 in each (Table 7).

**Table 6:** CD-HIT results of datasets

	Positive	Negative 1	N1-P	Negative 2
Original	1492	4433	–	2635
1.0	565	2753	2697	1585
0.9	398	2055	2559	1178
0.8	306	1664	2426	892
0.7	249	1358	2290	724
0.6	201	1097	2091	624
0.5	159	765	1667	531
0.4	107	439	1101	399

**Table 7:** Number of peptides in each dataset

	Positive	Negative 1	Negative 2
Training set	463	463	463
Testing set	100	100	500

### Features investigation

To utilize machine learning methods analyzing peptide sequences, features of sequences have to be extracted. In this research, 4 features are considered: amino acids composition (AAC), N5C5, k-space and PSSM.

### AAC

The AAC is the proportion of each amino acid in a given peptide sequence. It summarizes the peptide information in a vector of 20 dimensions. The AAC method has been successfully and widely applied in sequence-based classifications [29–32].

### N5C5

Five amino acids from both the N-terminal and C-terminal end of a given peptide are cut off and then connected as a novel sequence. Then the proportion of each amino acid in those new N5C5 sequences is calculated. Furthermore, to better analyze N5C5 sequences and visualize analysis results, heatmaps that show frequencies of each amino acid in each position are generated.

### K-space

The K-space method extracts pairs of amino acids that have k (k = 0, 1, …) spacing from a given peptide sequence. In total, (N-k-1) pairs are selected from a peptide sequence which consists of N amino acids. After gathering all amino-acid-pairs, the frequency of each kind of pair is counted. To explore k-space diversity between the positive dataset and those two negative datasets, the difference value of k-space frequency in the positive dataset and that in the negative datasets is then calculated. At last, those difference values of amino-acid-pairs are sorted, and ten pairs with the highest difference values are listed.

### PSSM

PSSM is generated from a group of sequences previously aligned according to structural or sequence similarity. A PSSM for a given protein is an N 20 matrix P = {Pij: i = 1… N and j = 1 … 20}, where N is the length of the protein sequence. It assigns a score P*ij* for the *j-*th amino acid in the *i-*th position of the query sequence. A large value indicates a highly conserved position while a small value indicates a weakly conserved position [33].

### Model construction by machine learning techniques

In this study, a supervised learning technique should be applied on text data for classification. Therefore, SVM [21] is utilized in cooperation with SMO [22]. For model construction, WEKA software (version 3.8.4) [22], and packages including LIBSVM (version 3.24) [23] and SMO package (using default parameters) within WEKA are utilized.

SVM is a data-driven supervised algorithm that constructs separating hyperplanes in high-dimensional space and selects the maximum-margin one for classification [34]. Based on its solid theoretical foundations, SVM has been successfully applied in various recognition and classification studies, including text classification [35], which is utilized in this research. SVM has also been successfully and widely used for high-dimensional biological data, including examination of gene expression profiles [36], mass spectra and genomics projects [37]. Comparing to other classifiers, such as artificial neural networks, SVM shows higher accuracy, particularly when the numbers of features are large [37]. Furthermore, to improve the performance of the SVM model, a program is designed to determine the optimum value of the weight vector for each model in this research. As for adjusting gamma and cost value, a program in the LIBSVM package [38] is applied to each model.

However, SVM does have some problems, including complexity and slow training speed for large-scale data. To solve these problems, another algorithm, SMO, is also applied for classification and shows both faster speed and better performance. SMO is a new algorithm for training SVMs, which breaks large quadratic programming (QP) optimization problem, a significant obstacle in the original SVM algorithm, into a series of smallest possible QP problem. By solving those smaller QP problems analytically, a time-consuming numerical QP optimization as an inner loop could be circumvented, and thus the computational time is shortened. The SVM maximization problem is as:

$$\mathop {\max }\limits_\lambda \sum\limits_{j = 1}^m {{\lambda _j}} - \frac{1}{2}\sum\limits_{j = 1}^m {\sum\limits_{k = 1}^n {{\lambda _j}} } {\lambda _k}{y_j}{y_k}{x_j}{x_k},\quad 0 \leq {\lambda _j} \leq C,\;\quad {\forall _j},\;\sum\limits_{j = 1}^m {{y_j}} {\lambda _j} = 0$$

where λ is the Lagrange multiplier, x is the input data and y represent the class label. In SMO, two Lagrange multipliers ${\lambda_1},{\lambda_2}$ are optimized while all the other multipliers are kept constant using this equation [39]:

$${\lambda_1}{y_1} + {\lambda_2}{y_2} = - \mathop \sum \limits_{j = 3}^m {\lambda_j}{y_j} = c.$$

Moreover, since SMO only utilizes linear amount of memory, it can handle very large training sets [22], which is perfectly aligned with the need in the biological data analysis. To compute a linear SVM, only one weight vector needs to be stored. The stored weight vector can be easily updated to reflect new Lagrange multiplier values by:

$${\vec w^{{\text{new}}}} = \vec w + {y_1}\left( {\alpha_1^{new} - {\alpha_1}} \right)\overrightarrow {x_1} + {y_2}\left( {\alpha_2^{new,clipped} - {\alpha_2}} \right)\overrightarrow {x_2} l.\,[23]$$

This algorithm has shown success in some biological applications, such as metabolism studies [40, 41], genomics [42] and molecular studies [43, 44].

### Parameter optimization

In the SVM model training process, the probability distribution of positive prediction and negative prediction are listed as P^+^ and P^−^. Then a weight, ranging from 0.1 to 10 with 0.1 as the interval, is multiplied to both P^+^ and P^−^. Classification is redone according to:

$$Result = \left\{ {\begin{array}{*{20}{c}} {positive,\,if\,weight \times {P^+ } \geq weight \times {P^- }} \\ {negative,\,\;if\,weight \times {P^+ } < weight \times {P^- }} \end{array}} \right.$$

In this way, the performance of the training model is changed. The weight that contributes to the highest accuracy of the training model is chosen as the final parameter and then be applied on the testing dataset, which leads to the final testing performance.

### Performance evaluation

**Fig. 6:**
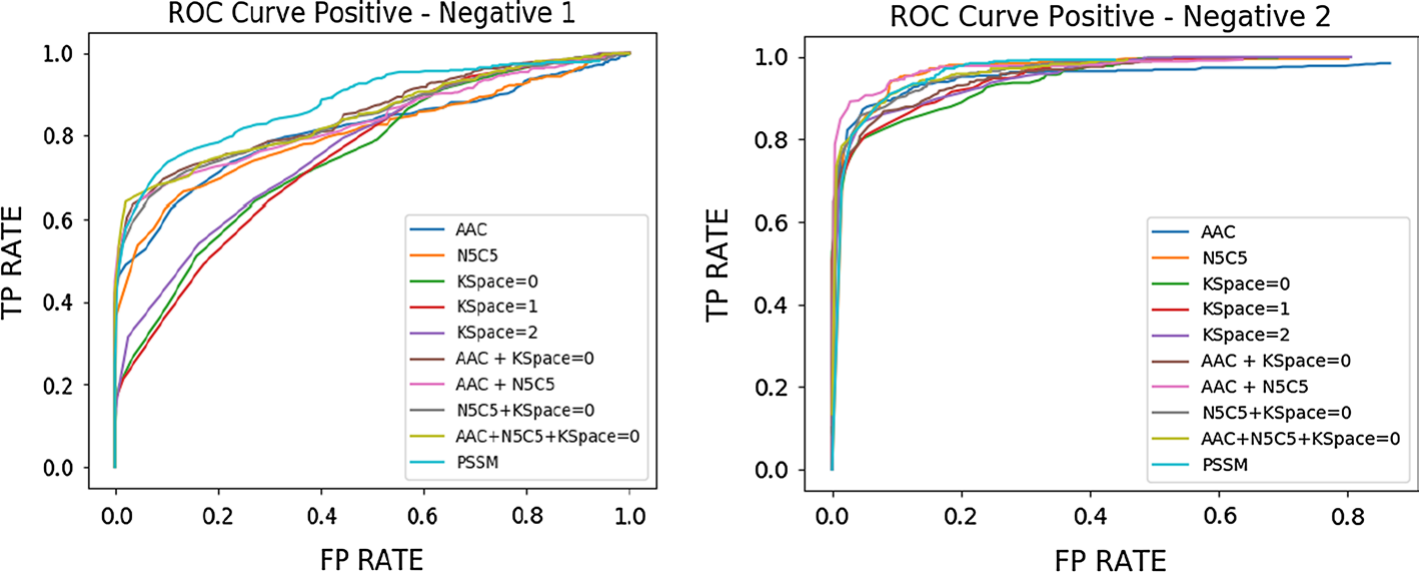
ROC curve for training models using SVM

To evaluate the performance of machine learning models, four indexes are calculated: accuracy, specificity (SP), sensitivity (SN) and Matthews correlation coefficient (MCC). Details of these metrics are shown as the following equations:

$${\text{SP}} = \frac{TN}{{TN + FP}}$$

$${\text{SN}} = \frac{TP}{{TP + FN}}$$

$${\text{ACC}} = \frac{TP + TN}{{TP + TN + FP + FN}}$$

$${\text{MCC}}\frac{TP \times TN - FP \times FN}{{\sqrt {\left( {TP + FP} \right)\left( {TP + FN} \right)\left( {TN + FP} \right)\left( {TN + FN} \right)} }}$$

where TP-true positive-represents the number of correctly predicted positive labels, TN-true negative-refers to the number of corrected predict negative labels, FP-false positive-represents the number of negative labels that are wrongly predicted as positive, and FN-false negative-refers to the number of positive labels that are wrongly predicted as negative by the classifier. In addition to those evaluation metrics, the receiver operating characteristic (ROC) curve (Fig. 6) is also generated in the step of weight adjustment to visualize the relationship of true positive rate and false positive rate, and used for comparison of performance.

### Cross-validation and independent testing sets

To test potential overfitting and evaluate the model, ten-fold cross-validation is applied in the model training step. Also, to evaluate the model built in this research and compare its performance with that of other existing tools, independent testing datasets are constructed in the dataset preparation step.

## Data Availability

The dataset generated and analyzed during the current study are available at: https://github.com/georginawan/proteomics_project.

## Abbreviations

AAC: Amino acid composition
ACP: Anticancer peptides
AMP: Antimicrobial peptides
PSSM: Position specific scoring matrix
SMO: Sequential minimal optimization
SVM: Support vector machine
